# The protective effects of Silymarin in diabetic wound healing: A review

**DOI:** 10.1097/MD.0000000000047801

**Published:** 2026-02-20

**Authors:** Hanghang Zhou, Chengqing Ding, Jianxiong Qiao, Qinyuan Wang, Xuanfen Zhang

**Affiliations:** a The Second Hospital and Clinical Medical School, Lanzhou University, Lanzhou, Gansu, China; b Department of Rehabilitation, The Armed Police Forces Hospital of Gansu, Lanzhou, Gansu, China; c Department of Plastic Surgery, The Second Hospital and Clinical Medical School, Lanzhou University, Lanzhou, Gansu, China.

**Keywords:** diabetes mellitus, diabetic wounds, phytotherapy, Silibinin, Silymarin, wound healing

## Abstract

Diabetic wounds are serious and challenging complications of diabetes mellitus (DM) and are characterized by impaired healing. Its pathogenesis is complex and involves a variety of physiological and pathological changes, including vascular dysfunction, neuropathy, impaired neuropeptide signaling, hyperglycemia, persistent infections, inflammation, oxidative stress, and an impaired immune response. Despite significant advances in the understanding of the pathogenesis of diabetic wounds, current treatment options remain limited and often yield unsatisfactory results. Thus, there is an urgent need for alternative approaches to enhance diabetic wound healing. Silymarin (SM) is a natural flavonolignans derived from the traditional medicinal plant *Silybum marianum* (L.) Gaertn, with silibinin/silybin (SB) as its primary active component, and has shown considerable therapeutic potential in both experimental and clinical studies. This review synthesizes high-quality, peer-reviewed research on the use of SM/SB for DM and its complications, and suggests that SM/SB may promote wound closure through its established anti-inflammatory, antioxidant, antidiabetic, hypoglycemic, neuroprotective, and vascular/endothelial-protective properties. In this review, we highlight the current beneficial modulatory effects of SM/SB on diabetic wounds and explore the potential mechanisms that may support these benefits. Although early evidence is promising, further high-quality clinical studies are needed to confirm the efficacy of SM/SB in diabetic wound healing. Additionally, advancements in biomaterials could enhance the in vivo efficacy of SM, accelerating the translation of SM/SB-based therapies into clinical practice and offering a novel, complementary treatment or an innovative alternative to conventional protocols for diabetic wound closure.

## 1. Introduction

Diabetes mellitus (DM) is a chronic metabolic disorder characterized by impaired glucose regulation and insulin resistance (IR), which poses a serious threat to human health and places a significant burden on healthcare systems worldwide. The prevalence of DM continues to rise. According to the latest diabetes data released by the International Diabetes Federation, the number of adult DM patients aged between 20 and 79 worldwide, which was 589 million in 2024, is projected to increase to 853 million in 2050.^[1]^ Prolonged hyperglycemia leads to widespread tissue and organ damage, starting with microvascular and neurological impairment and progressing to macrovascular pathologies and major organs, resulting in severe consequences. Among the most serious and costly complications are diabetic wounds, particularly diabetic foot ulcers (DFU), which are difficult to heal.^[2]^ It has been reported that the lifetime prevalence of DFU in diabetic patients may reach as high as 34%.^[3]^ As the prevalence of DM and the number of patients increases, the cases of diabetic wounds will also increase.^[4]^ Under normal circumstances, acute wound healing follows a predictable process consisting of 4 overlapping phases: hemostatic, inflammatory, proliferative, and remodeling.^[5]^ However, in DM, various mechanisms involved in these processes are disrupted, resulting in impaired wound healing. The pathogenesis of diabetic wounds is complex and involves factors such as hyperglycemia, chronic inflammation, oxidative stress, infections, microcirculatory disorders, vascular and endothelial dysfunction(ED), and impaired neuropeptide signaling, which contribute to delayed wound repair.^[6,7]^ Currently, commonly used treatment methods include glycemic control, antimicrobial therapy, surgical debridement, negative pressure therapy, advanced dressing, hyperbaric oxygen therapy, skin grafting, and amputation, often producing limited results with unsatisfactory prognoses.^[8,9]^ Managing diabetic wounds remains a significant clinical challenge.^[10]^ Thus, there is an urgent need for an effective, safe, and reliable treatment that can significantly accelerate the healing process in diabetic wounds.

SM, a natural blend of flavonolignans, is derived from the seeds of the medicinal plant *Silybum marianum* (L.) Gaertn.^[11]^ SB, a flavonolignan monomer, is the primary bioactive component of SM, accounting for approximately 60% of its abundance, with the molecular formula C25H22O10.^[12]^ SM/SB exhibits a spectrum of biological properties, including antioxidant, anti-inflammatory, anticancer, hepatoprotective, and cardiovascular protective effects.^[13,14]^ Clinically, SM is commonly used as an adjunctive therapy for liver damage such as cirrhosis and chronic hepatitis.^[13]^ Recently, SB has demonstrated potential therapeutic benefits for DM and its complications, including enhanced wound healing.^[15]^ Given these properties, SM/SB emerges as a promising natural plant extract for promoting diabetic wound healing. This review summarizes the key factors that influence wound healing in DM. The beneficial effects of SM/SB in accelerating diabetic wound healing were further discussed to thoroughly elucidate the underlying mechanisms and provide new insights for basic and clinical research in diabetic wound therapy.

## 2. Review methodology

This narrative review was conducted through a systematic search of relevant research articles published between January 1, 2010, and November 18, 2024, in the electronic databases PubMed, EMBASE, and CNKI. The following search strategy was used to identify potentially eligible publications: (((Silymarin [Title/Abstract]) OR (Silybin [Title/Abstract]) OR (Silibinin [Title/Abstract]))) AND (((((Diabetic Wound [Title/Abstract]) OR (Diabetic Wound Healing [Title/Abstract])) OR (Wound Healing [Title/Abstract])) OR (Skin Injury [Title/Abstract])) OR (Wound [Title/Abstract]))). Publications were then selected in a stepwise manner according to the following criteria: first, only articles published in English were considered; second, only original research articles were included; furthermore, studies related to cancer or other diseases were excluded. A total of 14 articles were ultimately included.

## 3. Overview of Silymarin

Phytotherapy ranks among the most esteemed complementary and alternative medical treatments globally.^[16]^ Silybum Marianum is a well-known plant that has both medicinal and nutritional properties.^[17]^ SM is a standardized extract from its seeds. It is a mixture of flavonolignans, the main components of which are SB, silicristin, isosilibinin, and silidianin, with SB being the predominant and most biologically active isomer.^[18]^ SM/SB possesses a range of biological activities, with its most prevalent and traditional use being the treatment of various liver and gallbladder ailments.^[19]^ Nevertheless, the therapeutic efficacy of SM/SB is undermined by its poor water solubility and bioavailability. Consequently, researchers have devised an array of solubilization systems, such as SM solid dispersion, SM phospholipid complex, SM sulfobutyl ether-β-cyclodextrin inclusion complex, and SM self-microemulsifying drug delivery systems, to effectively tackle this challenge.^[20]^ Due to their unique biological and pharmacological properties, SM/SB are being intensively studied. SM/SB has anti-inflammatory, antioxidant, antifibrotic, reactive oxygen species (ROS) scavenger effects, and promotes the repair and regeneration of hepatocytes to protect the liver, primarily by precise modulation and regulation of signaling pathways such as the nuclear factor kappa B (NF-κB) pathway,^[21]^ nuclear factor erythroid 2-related factor 2 (Nrf2) pathway,^[22]^ Wnt/β-catenin pathway,^[23,24]^ phosphatidylinositol 3-kinase/protein kinase B/mechanistic target of rapamycin (PI3K/Akt/mTOR) pathway,^[23]^ sirtuin pathway,^[25]^ and mitogen-activated protein kinase (MAPK) signaling pathway.^[26]^

In addition, numerous studies have documented the antineoplastic effect of SM in hepatocellular carcinoma, breast cancer, glioblastoma, gastrointestinal tumors, lung cancer, prostate cancer, and other common tumors.^[27,28]^ SM/SB achieves its anticancer activity primarily by inducing cell cycle arrest at the G1/S transition, activating inhibitors of cyclin-dependent kinases, reducing the formation of antiapoptotic gene products, and down-regulating inflammatory transcription factor pathways.^[27]^ It has now been reported that SB-based cancer therapies are currently in clinical trials.^[29]^

Moreover, SM/SB has cardiovascular protective,^[13]^ antiviral,^[30]^ photoprotective,^[31]^ and skin conditioning properties.^[32]^ Al-Rasheed et al^[33]^ demonstrated that SM, whether used independently or in conjunction with chlorogenic acid and/or melatonin, effectively mitigated cardiac toxicity induced by carbon tetrachloride in a rat model of cardiomyopathy. SB treatment reduces myocardial cell apoptosis, alleviates mitochondrial damage, endoplasmic reticulum (ER) stress, the generation of ROS, neutrophil infiltration and cytokine release, thereby exerting a protective effect against cardiac dysfunction induced by myocardial ischemia/reperfusion injury.^[34]^ Studies have shown that SB can inhibit the function of the RNA-dependent RNA polymerase of hepatitis C virus independently of the intracellular interferon-induced antiviral pathway, thus suppressing viral replication.^[11]^ This is consistent with clinically observed effectiveness. Oral administration of silymarin-vitamin E phospholipid complex tablets and ribavirin for 12 months in patients with chronic hepatitis C significantly reduced viral load.^[35]^ For dermatological conditions, SM/SB has been shown to be beneficial for various skin issues, including photodamaged skin, atopic dermatitis, melasma, facial acne, rosacea, and psoriasis. With regard to photoaging of the skin, for example, Vostálová et al^[31]^ investigated the protective role of SM and its flavonolignans against photoaging and found that they safeguard the skin from the detrimental impacts of solar radiation by scavenging free 1,1-diphenyl-2-picryl-hydrazyl radical radicals and have anti-collagenase and anti-elastase properties. In vitro, pretreatment of primary human skin fibroblasts with SM/SB followed by UVA exposure significantly reduced UVA-induced ROS generation and single-strand break formation.^[36]^

Recent studies have indicated the therapeutic potential of SM/SB, in the management of DM and its complications. For instance, in a streptozotocin (STZ)-induced diabetic rat model, SB treatment prevented the decrease in insulin levels and ameliorated hyperglycemia.^[37]^ In the same model, SB demonstrated neuroprotective potential in diabetic peripheral neuropathy (DPN) through SIRT1 activation and antioxidant mechanisms.^[38]^ In a review by MacDonald-Ramos et al,^[39]^ the authors noted that SM enhances insulin sensitivity, reduces insulin resistance (IR), and improves carbohydrate and lipid metabolism. A clinical cohort study suggested that silymarin might serve as a therapeutic alternative for improving IR in nondiabetic obese patients.^[40]^ In a study on retinal microvascular damage in diabetic rats, SM exerted a protective effect against diabetic retinopathy, potentially through suppression of the AGEs/RAGE axis.^[41]^ Furthermore, research by Liu et al^[42]^ confirmed that SB alleviates renal fibrosis in vitro and in vivo via inhibition of the NF-κB pathway, indicating that SB may represent a potential agent for mitigating renal fibrosis in diabetic nephropathy. Additionally, in vivo studies have shown that SB supplementation prevents bone loss in diabetic rats, thereby helping to maintain bone health.^[43]^

**Figure 1.**
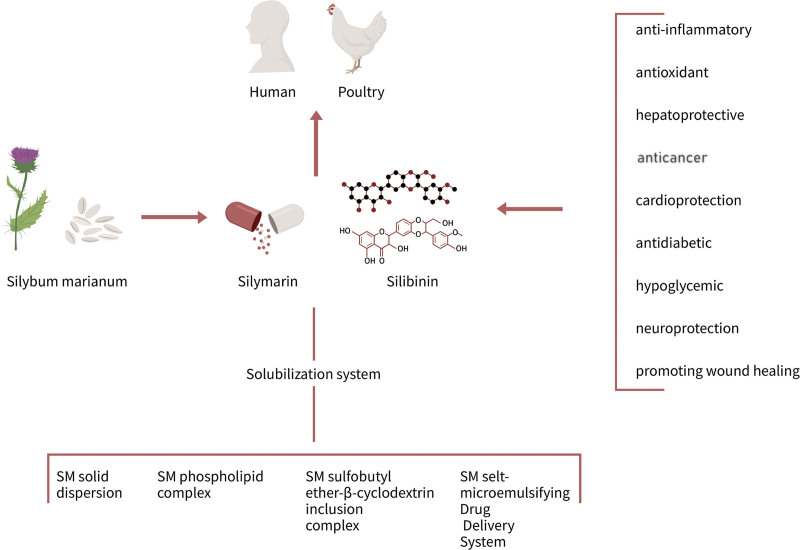
The natural sources, main components, biological and pharmacological properties of SM, and the developed solubilization systems. SM = silymarin.

In summary, SM/SB, as a promising medicinal plant extract, exhibits multiple biological activities and pharmacological effects, and its administration has been demonstrated to be safe. With advances in biotechnology and material science, issues related to its water solubility and bioavailability are expected to be effectively addressed (Fig. 1). This review focuses specifically on the beneficial effects of SM/SB on wound healing under hyperglycemic conditions and the underlying mechanisms, which will be discussed in detail in the following sections.

## 4. Overview of diabetic wounds

**Figure 2.**
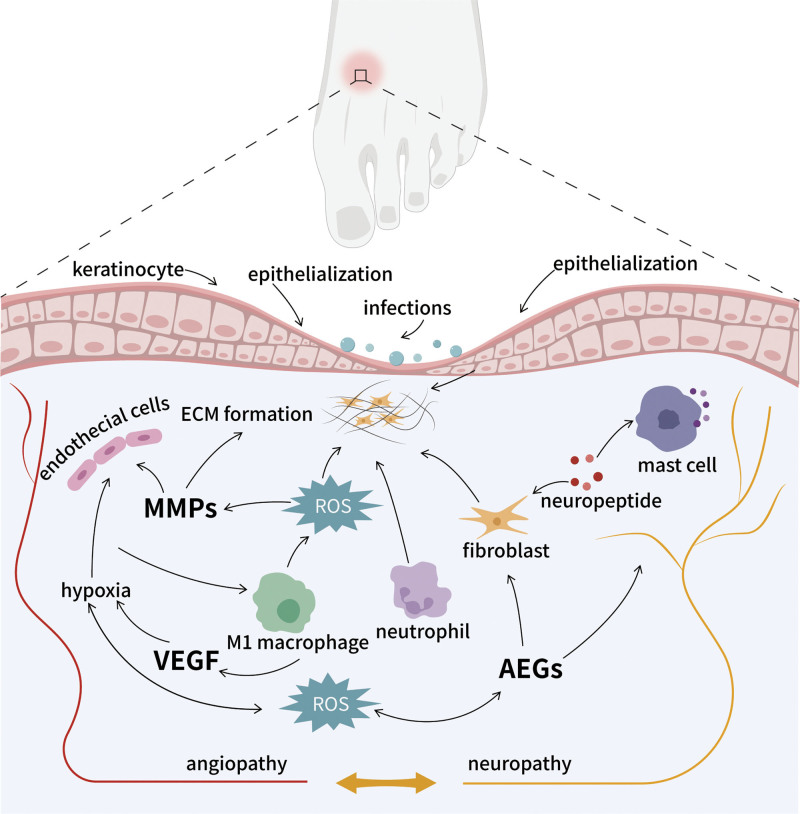
The main pathophysiological factors affecting the healing of diabetic wounds.

Diabetic wounds are a serious complication of DM, characterized by prolonged treatment duration, diminished quality of life, and exorbitant treatment costs. These factors cause significant physical and psychological distress for patients and impose a substantial financial burden on families and society.^[44]^ The pathogenesis of diabetic wounds is complex and not fully understood. However, numerous studies have confirmed that vascular dysfunction, neuropathy, disrupted neuropeptide signaling, hyperglycemic stimulation, persistent infections, inflammation, oxidative stress, and compromised immune response significantly affect diabetic wound healing (Fig. 2). As we all know, wound healing is an intricate and dynamic process, meticulously coordinating the phases of hemostasis, inflammation, proliferation, and remodeling to ultimately restore skin structure and function.^[45]^ Diabetic wound healing mainly involves keratin-forming cells, fibroblasts, endothelial cells, macrophages, neutrophils, and associated deposition and remodeling of the extracellular matrix.^[46]^ Acute wounds usually heal rapidly and completely. DM affects all stages of wound repair, leading to an abnormally amplified inflammatory stage, which is mainly caused by hyperglycemia, oxidative stress, impaired angiogenesis, and excessive production of pro - inflammatory cytokines.^[47]^

Angiogenesis is closely related to the provision of oxygen and nutrients necessary for wound closure, and is a crucial aspect of the proliferative phase of wound healing.^[48]^ Vascular dysfunction, impaired macrophage phenotype switching, perfusion defects, and increased AGEs caused by persistent hyperglycemia in diabetic wounds lead to difficulties in regeneration of wound vasculature and delayed healing. VEGF is deemed a pivotal regulator in the onset of wound healing, fostering epithelialization, collagen deposition, and angiogenesis.^[49]^ Research has shown that VEGF expression levels in diabetic wounds are significantly reduced compared to those in normal wounds.^[50]^ However, throughout the recuperative phase of diabetic wounds, the transformation of the macrophage phenotype is impaired, resulting in impaired conversion of the M1 (pro-inflammatory phenotype) to the M2 (anti-inflammatory phenotype) and reduced release of proangiogenic mediators such as VEGF, resulting in reduced angiogenesis and excessive inflammatory reaction in diabetic wounds. It has been demonstrated that VEGF directly induces polarization of M2 macrophages in vitro, thereby promoting DFU healing.^[49]^ In addition, cellular pyroptosis and ferroptosis-induced ED in the high-glucose microenvironment exacerbate the progression of vasculopathy.^[51,52]^ Additionally, in hyperglycemic conditions, sugars and proteins form AGEs, which could impede the healing of diabetic wounds by inhibiting the proliferation of keratinocytes and fibroblasts, altering the structure of collagen and keratin, and compromising blood flow to the affected areas.^[53]^

Inflammation and oxidative stress generating excessive ROS is a distinguishing feature of diabetic wounds. Diabetic wound healing becomes arrested in the inflammatory phase and is abnormally prolonged.^[54]^ Bacterial invasion of the wound can release toxins and proteases, leading to delayed collagen synthesis and epithelialization of the wound margins.^[55]^ Furthermore, biofilms formed by bacteria adhering to wounds can evade host immune cells, resulting in poor infection control and chronic inflammation. Evidence suggests that in diabetic wounds, the persistent recruitment of macrophages and neutrophils creates a microenvironment abundant in pro-inflammatory cytokines and ROS, thereby inhibiting the proliferation of fibroblasts and keratinocytes and impairing the healing process.^[56]^ Additionally, ROS and pro-inflammatory cytokines in diabetic wounds contribute to the persistently high expression of matrix metalloproteinases and the degradation of extracellular matrix (ECM) and growth factors essential for wound repair, leading to delayed healing.^[57]^

DPN is another important element in the development of diabetic wounds with delayed healing.^[58,59]^ The pathogenesis of DPN may be associated with chronic neuronal ischemia and hypoxia, inflammation, and oxidative stress caused by hyperglycemic stimulation and microcirculatory disorders.^[60,61]^ Moreover, peripheral nerve fibers in the skin, upon injury, become stimulated and release various neuropeptides into the wound microenvironment.^[62]^ Reduced expression of these neuropeptides has been demonstrated in diabetic patients. Furthermore, changes in their expression and function can lead to impairment of diabetic wound healing.^[58]^ Neuropeptides, including neuropeptide Y, substance P, and calcitonin gene-related peptide, are neuropeptides involved in the regulation of the immune response and wound healing. They have been demonstrated to affect vascular endothelial cells, macrophages, fibroblasts, keratinocytes, and endothelial progenitor cells, thereby influencing inflammation and angiogenesis during the wound healing process.^[63]^ In conclusion, the treatment of diabetic wounds is a challenging clinical problem owing to its complex pathogenesis. Therefore, developing novel strategies for diabetic wound healing is clinically important and socially valuable for accelerating healing.

## 5. Beneficial effects and potential mechanisms of Silymarin on diabetic wounds

### 5.1. Protective function against anti-inflammatory and oxidative stress

Excessive production of ROS and oxidative stress, induced by the hyperglycemic state in diabetic wounds, significantly hinders the healing process.^[64]^ SM and its primary bioactive constituent, SB, have demonstrated antioxidant and anti-inflammatory effects in various in vitro and in vivo models.^[65]^ In vitro studies have shown that SM/SB exerts anti-inflammatory effects in macrophages,^[66]^ monocytes,^[67]^ epithelial cells,^[68]^ human skin fibroblasts,^[69]^ keratinocytes,^[70]^ human chondrocytes,^[71]^ connective tissue cell,^[72]^ and cancer cells.^[65]^ The anti-inflammatory action of SM/SB is thought to involve the modulation of several signaling pathways, including NF-κB, MAPK, PI3K/Akt, NAD/SIRT2, and mTOR/NF-κB.^[65]^ In a mouse model of experimental nonalcoholic steatohepatitis (NASH), in vivo activation of the caspase 8 and Fas-associated protein with death domain-like apoptosis regulator-c-Jun N-terminal kinase pathway by silymarin mitigated oxidative stress by enhancing antioxidant enzyme activities while concurrently inhibiting pro-oxidative enzyme activities.^[73]^ NF-κB is known to be a classic inflammatory pathway. SM inhibits the O-GlcNAcylation-dependent NF-κB signaling pathway, reducing TNF-α, IL-6, and inducible nitric oxide synthase (iNOS) expression, thereby reducing inflammation and improving liver function in NASH models.^[74]^ Furthermore, the study by Wang et al^[75]^ has demonstrated that mesenchymal stromal cells modified with nanoparticles loaded with SB reduce the intracellular ROS level by activating the Nrf2/ARE signaling pathway. This enhances the survival rate of mesenchymal stromal cells after their transplantation into full-thickness skin wounds and improves the process of wound healing.

Notably, SM/SB is recognized for its powerful antioxidant capacity, mainly achieved by inhibiting key antioxidant enzymes and preventing free radical-induced DNA damage.^[76]^ For example, SM can directly diminish ROS levels via the Nrf2 signaling pathway and inhibit NF-κB signaling activity, thus mitigating hepatic oxidative damage induced by ROS.^[21]^ Moreover, the oxidation of free fatty acids can result in elevated levels of ROS and RNS, thereby triggering inflammatory pathways. SM significantly alleviated the inflammatory response by inhibiting the expression of iNOS.^[76]^ Furthermore, when oxidized DNA enters the cytoplasm, it can induce the expression of stimulator of interferon genes (STING), thereby triggering the Interferon-beta and inflammatory cascade reactions.^[77]^ The intervention of silybin can block this process and down-regulate the downstream STING-mediated neuroinflammation by reducing ferroptosis damage.^[77]^

### 5.2. Antidiabetic activity

Pancreatic β-cell dysfunction, which leads to reduced insulin secretion, is a major contributor to DM. Research indicates that ER stress in pancreatic β-cells is intricately connected to the impairment of pancreatic exocrine function and the development of DM.^[78,79]^ Chronic hyperglycemia and high fatty acids can cause glucotoxicity and lipotoxicity in pancreatic β-cells, triggering ER stress, inflammation, and mitochondrial stress, resulting in reduced insulin gene expression, impaired insulin secretion, and increased apoptosis.^[78]^ The antidiabetic properties of SM have been extensively studied and confirmed.^[80–82]^ Sahin et al^[83]^ found that SM (200 mg/kg) normalized lipid profiles, AST, ALT, glucose levels, and ER stress markers in a mouse model of nonalcoholic fatty liver disease (NAFLD), suggesting its potential for treating NAFLD and other diseases related to ER stress. Feng et al^[84]^ demonstrated that SM ameliorates the disordered glucose metabolism of mice with diet-induced obesity by activating the hepatic silent information regulator-1 pathway. In addition, in the rat insulinoma cell line-1 (INS-1) cells of the rat pancreatic β-cell line, islet amyloid protein and amyloid-β can induce the production of ROS and reactive nitrogen species (RNS), ultimately leading to cell death.^[85]^ SB exerts a protective effect on INS-1 cells by down-regulating ROS/RNS through the activation of estrogen receptor phosphorylation.

Interestingly, estrogen may be involved in the pathogenesis of T2DM and exhibits sex differences, with postmenopausal estrogen reduction accelerating the progression of IR and DM.^[86]^ Targeting estrogen could offer a promising new approach to regulate glucose metabolism and prevent diabetes. Chu et al^[87]^ reported that SM (100 mg/kg/day) markedly reduced pro-inflammatory cytokines (TNF-α and IL-1β) and enhanced insulin secretion by up-regulating estrogen receptor-α (ER-α) expression in a high-fat diet-induced diabetes model. This effect likely involves activation of the Nrf2 antioxidant pathway, which elevates the expression of Nrf2 and its downstream effector, heme oxygenase (HO)-1, in pancreatic β-cells of diabetic rats. This activation inhibits ROS production, thereby protecting rat insulinoma cells from high-glucose-induced cytotoxicity and dysfunction.

IR and obesity are major risk factors for T2DM. Therefore, weight control and improvement in IR are key strategies for treating T2DM and its complications. Numerous studies have supported the role of SM/SB in reducing insulin resistance. In a high-fat diet-induced obesity model, dietary supplementation with SM (40 mg/100 g) significantly improved fasting blood glucose levels, insulin resistance, and hyperlipidemia and alleviated inflammation by mediating the hepatic Farnesyl-X receptor signaling pathway.^[88]^ A longitudinal study of 6 insulin-resistantobese women showed that after 12 weeks of SM soft gel administration (equivalent to 45 mg SB), fasting plasma glucose and homeostatic model assessment for insulin resistance index (HOMA-IR) significantly reduced.^[40]^ Furthermore, a recent systematic review and meta-analysis encompassing 26 randomized controlled trials with 2375 participants demonstrated that SM significantly lowered non-HDL and HOMA-IR levels, elevated HDL levels, and ameliorated insulin resistance and NAFLD.^[89]^

### 5.3. Neuroprotective function

DM has been reported to cause neurological damage, including DPN, affecting approximately 10% to 15% of diabetic patients.^[90]^ Currently, little is known about the pathogenesis of diabetic neuropathy. Nevertheless, Sloan et al^[91]^ reported the pathogenesis of diabetic sensorimotor peripheral neuropathy, which may be related to neuronal inflammation, oxidative stress, mitochondrial dysfunction, and cell death due to hyperglycemia, dyslipidemia, and microangiopathy. Normal innervation is essential for successful wound closure. Denervation studies provide evidence that cutaneous innervation functions in wound healing and reversal of neuropathy have been shown to facilitate wound healing in DM.^[58]^ In an animal model of STZ-induced DPN, a 2 weeks of SM treatment improved neurobehavioral symptoms in DPN rats through activation of sirtuin 1, increased mitochondrial autophagy, and counteracted oxidative damage caused by high-glucose-mediated neurotoxicity through upregulation of Nrf2.^[38]^ In recent years, SM has been regarded as a highly promising neuroprotective therapy and can be used to treat a variety of neurological diseases, including Parkinson disease, Alzheimer disease, and diseases associated with cerebral ischemia.^[92]^

Similarly, SB has been shown to have a protective role against neurons in vitro. Wang et al^[93]^ reported that SB protected cortical neuronal cells from oxidative damage during ischemia-reperfusion and prevented neuronal apoptosis. The underlying mechanism involves the inhibition of autophagy through upregulation of the PI3K/Akt signaling pathway, which suppresses the expression of microtubule-associated protein 1 light chain 3 and Beclin-1 under oxidative stress. Li et al^[94]^ confirmed the protective effect of SB on neurons subjected to hypoxia/reoxygenation. Treatment of N2a cells with varying concentrations of SB (5, 10, 20, and 50 μM) for 3h significantly reduced hypoxia/reoxygenation-induced damage, and alleviated oxidative stress and mitochondrial dysfunction. In addition, Alzheimer disease and Parkinson disease are considered neurodegenerative disorders, and SB has been shown to provide neuroprotection against neurodegenerative lesions. Liu et al^[77]^ revealed that SB promoted hippocampal neuron survival in mice by down-regulating p53-mediated iron death while reducing stimulator of interferon gene-mediated neuroinflammation and cognitive dysfunction in rats with Alzheimer disease. In an experimental model of Parkinson disease, SB mitigated mitochondrial dysfunction and selective death of dopaminergic neurons by stabilizing the mitochondrial membrane potential.^[95]^

Furthermore, SB improved learning and memory in mice with STZ-induced dementia by inhibiting the hyperphosphorylation of tau proteins and suppressing neuronal cell apoptosis.^[96]^ The mechanism of this neuroprotection may be that the impaired insulin signaling pathway is improved by inhibiting the hyperphosphorylation of tau protein, which increases the expression of insulin receptor and insulin-like growth factor-1 receptor, thereby inhibiting neuronal apoptosis. Oxaliplatin is an organic platinum-based drug with anticancer properties that is commonly used in chemotherapy for colorectal cancer, in which the most common side effect is the induction of oxidative damage to the nervous system. SB effectively alleviates oxaliplatin-dependent pain by down-regulating the oxidation of lipids, proteins, and DNA levels in neural tissue in a model of oxaliplatin-induced experimental neuropathy.^[97]^

### 5.4. Hypoglycemic function

Evidence suggests that SM may act as a hypoglycemic agent independent of increasing plasma insulin concentrations.^[98]^ SM supplementation improves the glycemic index and lipid profiles in T2DM patients. The underlying mechanisms of hypoglycemia may be associated with antioxidant properties, increased endogenous antioxidants, inhibition of Protein Kinase C and MAPK signaling pathways, suppression of gluconeogenesis, and downregulation of nuclear receptor gene expression.^[99]^ However, a recently updated randomized clinical trial reported different results, with no statistically significant changes in the glycemic index or lipid profiles between baseline and the conclusion of the study.^[100]^ This discrepancy may be due to uncontrolled confounding factors or insufficient sample size, highlighting the need for further studies with larger samples to validate the effect of SM on glucose metabolism in T2DM.

### 5.5. Protects endothelium and promotes angiogenesis

Angiogenesis effectively promotes wound closure; however, diabetic wounds often suffer from impaired angiogenesis and vascular integrity/ED, both of which are major contributors to nonhealing wounds. Studies have shown that diabetic wounds exhibit significantly delayed neovascularization, reduced blood vessel surface, fewer branch connections, and shorter vessel lengths.^[101]^ Despite the limited literature on the effects of SM/SB on the vascular endothelium and angiogenesis, some valuable studies exist. ED is characterized by cardiovascular disease in patients with diabetes, and elevated levels of asymmetric dimethylarginine are strongly associated with cardiovascular ED. Volti et al^[102]^ found that administration of SB (20 mg/kg/day, 4 weeks) improved endothelium-dependent vasodilation in response to acetylcholine and IR in db/db mice. Additionally, dysregulated extracellular signal-regulated kinase (Erk)-5 can lead to ED; however, SM helps maintain endothelial homeostasis through its antioxidant properties and by increasing Erk-5 expression. This effect reverses ED induced by oxidized low-density lipoprotein (ox-LDL) and improves body weight, glycemia, LDL, and ox-LDL levels in obese mice.^[103]^ Interestingly, the study also revealed the phytoestrogenic properties of SM, and SM treatment significantly improved vascular and endothelial function in older ovariectomized rats.^[104]^ In addition, researchers have reported the synthesis of a new zinc-silibinin complex that promotes angiogenesis by increasing VEGF and Ang1 in mouse mesenchymal stem cells. This complex also exhibits strong antimicrobial activity against *Escherichia coli* and *Staphylococcus aureus*.^[105]^

Reduction of VEGF in diabetic wounds impairs peripheral angiogenesis. In contrast, the increased VEGF-mediated proangiogenic response is unusually heightened in diabetic retinopathy and in the brains of diabetic patients, leading to immature, pathological vessels and predisposition to increased vascular permeability and hemorrhagic events.^[106]^ Glycogen synthase kinase-3β (GSK-3β) plays a crucial regulatory role in VEGF expression and angiogenesis.^[107]^ SM inhibits VEGF expression via GSK-3β in a concentration-dependent manner, thereby enhancing diabetes-induced proangiogenic responses in brain endothelial cells.^[108]^ Currently, numerous publications have reported the protective role of SM/SB against diabetic retinopathy through anti-VEGF mechanisms. Interestingly, angiogenesis varies significantly among diabetic wounds, diabetic retinopathy, and the diabetic brain in individual cases, and the underlying mechanisms remain unclear, necessitating further investigation.

### 5.6. Accelerated wound healing

**Figure 3.**
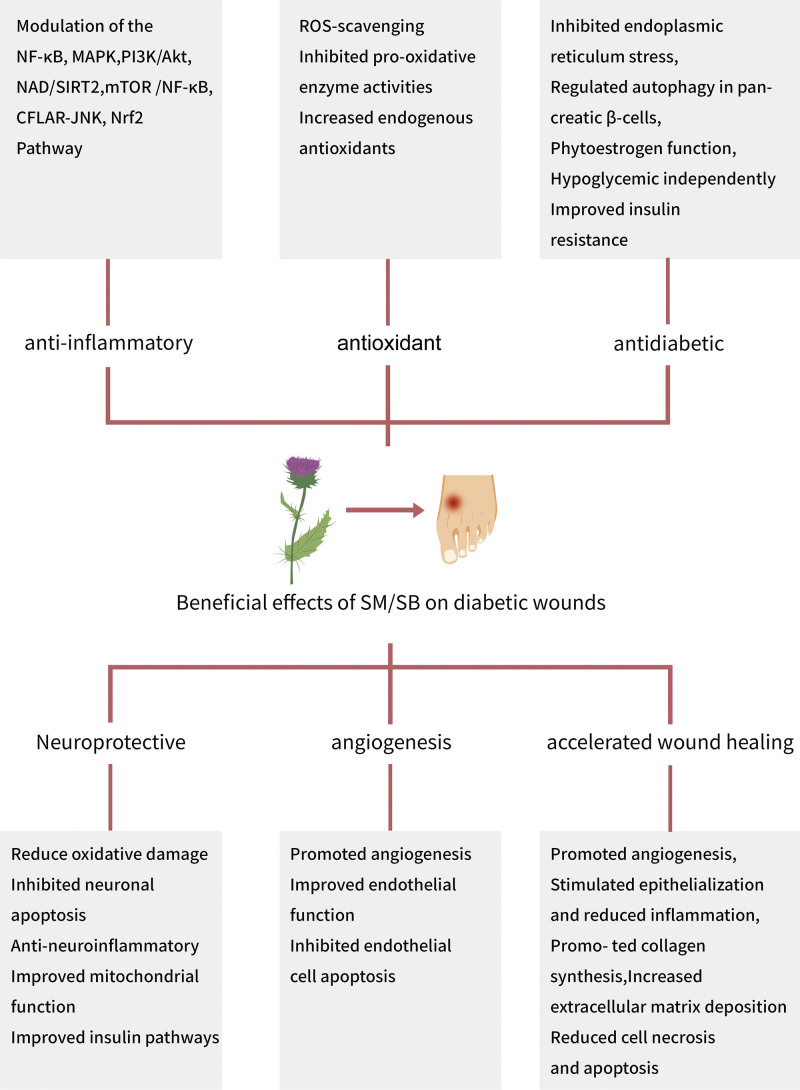
SM/SB exhibits beneficial effects against diabetic wounds by peer-reviewed studies. SB = silibinin/silybin, SM = silymarin.

Despite clinical attempts to manage diabetic wounds and promote their closure, the outcomes are often unsatisfactory and sometimes lead to severe complications. SM is a traditional medicinal plant extract with tremendous therapeutic potential based on experimental and clinical investigations. Previously, we have summarized the beneficial modulatory effects of SM/SB on diabetic wounds (Fig. 3). Although SM/SB has not been applied directly to diabetic wounds, several animal and in vitro studies have demonstrated its ability to accelerate wound healing (Table 1). Sharifi et al^[109]^ investigated that topical application of SM significantly reduced inflammatory cell infiltration and promoted epithelialization in full-thickness wounds in albino rats. In another study, the wound-healing activity of 0.2% SM gel was examined using a mouse model of incision and excision wounds. After 8 days of treatment, SM gel increased collagen synthesis, angiogenesis, fibroblast number, and wound tensile strength in granulation tissue while simultaneously reducing macrophage infiltration and significantly accelerating wound healing.^[110]^ Furthermore, Tabandeh et al^[111]^ reported that SM treatment (10% or 20%) accelerated wound closure in a dose- and time-dependent manner by promoting stromelysin-1 gene expression, collagen production, and remodeling of the extracellular matrix components. SM administration also ameliorated nitrogen mustard-induced skin damage in mice by reducing epidermal thickness, keratosis pilaris, necrosis, and apoptosis through mechanisms linked to DNA repair, inflammation, and oxidative stress pathways.^[112]^

**Table 1:** Characteristics of included studies demonstrating the beneficial effects of SM/SB on wound healing.

Experiment model	Drug administration	Concentration/dose	Duration	Effects and mechanism of action	References
Full-thickness skin wounds in albino rats	Local application	30 mg/kg	15 d	1. Accelerated wound healing 2. Stimulated epithelialization and reduced inflammation	Sharifi et al^[109]^
Incision and full-thickness wound in mice	Local application	0.2% gel	8 d	1. Accelerated wound healing 2. Reduced inflammatory cell infiltration 3. Promoted angiogenesis 4. Promoted collagen synthesis 5. Increased tensile strength of the wound	Samanta et al^[^^110^^]^
Full-thickness skin wounds in rats	Local application	10% or 20%	30 d	1. Accelerated wound healing 2. Increased STM1 gene expression 3. Increased extracellular matrix deposition	Tabandeh et al^[111]^
Nitrogen mustard-induced skin damage in SKH-1 mice	Local application	1 or 2 mg	24, 72 or 120 h	1. Reduced skin damage 2. Effected inflammation, oxidative stress, DNA damage, blister-associated pathways 3. Reduced cell necrosis and apoptosis 4. Reduced epidermal exfoliation and hyperkeratosis	Jain et al^[^^112]^
Soulful mustard-stimulated HaCaT cells	Mixed with culture medium	10, 50, and 100 µM	24 h	1. Reduced cell necrosis and apoptosis 2. Reduced production of IL-6, IL-8	Balszuweit et al^[113]^
LPS-stimulated human neonatal foreskin fibroblast cells	Mixed with culture medium	4.5, 9, 18, 36 µg/mL	24 h, 48 h	1. Increased anti-inflammatory and antioxidant capacity 2. Suppressed the LPS-induced mRNA expression	Sharifi et al^[1^^14^^]^

LPS = lipopolysaccharide, SB = silibinin/silybin, SM = silymarin.

In vitro studies have shown that the water-soluble prodrug silibinin-bis-succinct protected human keratinocyte HaCaT cells from the toxic effects of sulfur mustard and significantly reduced cell necrosis in a concentration-dependent manner.^[113]^ Similarly, SM protected human fibroblasts from lipopolysaccharide-induced inflammation and oxidative stress without affecting cell viability and proliferation.^[114]^ These findings suggest SM’s therapeutic potential of SM in wound healing by leveraging its multifaceted biological attributes to expedite wound healing by enhancing collagen deposition, angiogenesis, granulation, anti-inflammatory effects, epithelialization, and wound contraction. Although SM/SB appears promising for diabetic wounds, more high-quality evidence is needed to confirm its effectiveness.

## 6. Conclusions and prospects

This review is based on accessible, high-quality, peer-reviewed literature, suggesting that SM and its key bioactive constituents may be a potential, promising adjunctive, or alternative approach to conventional treatment for diabetic wounds. Their well-documented anti-inflammatory, antioxidant, ROS-scavenging, antidiabetic, hypoglycemic, neuroprotective, angiogenic, and endothelial-protective properties make SM/SB a potential candidate for treating diabetic wounds. Additionally, SM/SB is a cost-effective, eco-friendly, and safe traditional medicinal plant product, presenting a valuable source for targeted therapies for various ailments compared with current medications.

The pathogenesis of diabetic wounds is complicated, and no specific intervention is universally effective. However, evidence supports SM’s underlying the beneficial effects mechanisms and its major bioactive components in accelerating wound healing through attenuating inflammatory infiltrates, promoting angiogenesis and collagen deposition, and increasing epithelialization. Further research into the precise mechanisms of diabetic wound healing will significantly deepen our understanding of these conditions. However, there is a lack of direct evidence that SM/SB promotes healing of diabetic wounds. To confirm this and elucidate the molecular mechanisms, further high-quality basic and clinical studies are necessary to investigate their effects on granulation tissue, ECM, and epithelialization in the diabetic wound microenvironment, rather than overemphasizing clinical effectiveness.

Although our findings remain considerably distant from clinical application, several strategic efforts could facilitate future translation into clinical practice. First, more in-depth basic research – including cellular experiments, animal models, and functional validation – is needed to address the current mechanistic gaps and elucidate the dose-response relationship of silymarin in diabetic wound treatment. Second, given silymarin inherent limitations such as poor water solubility, low bioavailability, and short local retention time, it is essential to develop novel, biocompatible, and operable drug delivery systems. Priority should be given to topical formulations, including hydrogel-based materials, electrospun nanofiber membranes, and microemulsion preparations. Furthermore, comprehensive safety evaluations will be crucial to support subsequent clinical validation studies. Recently, a newly developed SM-loaded nanotransferrin hydrogel significantly reduced hyperglycemia compared to conventional drug delivery methods.^[115]^ Similarly, SB-loaded nanocapsule hydrogels showed more significant anti-inflammatory effects in irritant contact dermatitis in mice.^[116]^ Therefore, novel biomaterials incorporating SM and its bioactive components are expected to represent a promising complementary treatment or an innovative strategy to conventional treatment protocols for diabetic wound closure.

## Acknowledgments

We would like to thank Editage for the English language editing.

## Author contributions

**Conceptualization:** Hanghang Zhou, Xuanfen Zhang.

**Investigation:** Chengqing Ding.

**Methodology:** Hanghang Zhou.

**Software:** Qinyuan Wang.

**Visualization:** Qinyuan Wang.

**Writing – original draft:** Hanghang Zhou, Chengqing Ding, Jianxiong Qiao.

**Writing – review & editing:** Xuanfen Zhang.
